# Panuveitis with extensive retinal arteritis after delayed referral of retained lens fragments

**DOI:** 10.1016/j.ajoc.2026.102658

**Published:** 2026-09-02

**Authors:** Jun Young Park

**Affiliations:** Department of Ophthalmology, Uijeongbu Eulji Medical Center, Eulji University School of Medicine, Uijeongbu, South Korea

**Keywords:** Retained lens fragments, Retinal arteritis, Panuveitis, Phacoantigenic uveitis, Vitrectomy

## Abstract

**Purpose:**

To report severe panuveitis with extensive retinal arteritis caused by prolonged retained lens fragments following cataract surgery, in the setting of delayed referral during the 2024 Korean medical crisis.

**Observations:**

A 61-year-old woman presented with severe ocular pain and injection in the right eye two months after phacoemulsification cataract surgery performed at an outside hospital. The patient was unaware of any intraoperative complications, and no formal referral had been made. Vision was hand motion with elevated intraocular pressure (34 mmHg), hypopyon, and dense vitreous opacity suggesting panuveitis or endophthalmitis. Emergency 25-gauge pars plana vitrectomy was performed, during which retained lens particles were identified at the inferonasal periphery and removed using Ocutome® lensectomy. Intraoperative fundus examination revealed retinal arteries accompanied by granular, non-continuous peri-arterial precipitates. Correspondingly, fluorescein angiography demonstrated arterial beading, luminal stenosis, and peripheral non-perfusion, while optical coherence tomography confirmed hyperreflective thickening of the arterial wall consistent with arteritis. Following surgical removal of the antigenic lens material and oral corticosteroid therapy, ocular inflammation subsided, and the best-corrected visual acuity improved to 0.5 at 6-month.

**Conclusions and importance:**

Prolonged lens retention can cause panuveitis with chronic phacoantigenic inflammation extending to retinal vessels, resulting in extensive arteritis. Early vitreoretinal referral after retained lens fragments is critical, particularly during healthcare crises requiring prioritized communication to prevent vision-threatening sequelae.

## Highlights

• Delayed referral after retained lens fragments can present as painful panuveitis in the form of phacoantigenic uveitis.
• Extensive retinal vasculitis can manifest primarily as arterial sheathing and peri-arterial precipitates.
• Early vitrectomy is crucial for inflammation resolution and vision preservation.

## 1 Introduction

Cataract surgery is the second most common surgical procedure performed in the United States annually^1^ and ranks as the number one most frequently performed surgery in South Korea.^2^ Despite its high volume and established safety profile, retained lens fragments (posterior capsule rupture with lens fragment dislocation into the vitreous) occurs in approximately 1-2% of cases, potentially leading to serious complications if not promptly managed.^3^ Common sequelae include elevated intraocular pressure from retained lens material, corneal edema, cystoid macular edema, retinal detachment, and lens-induced uveitis due to phacoantigenic reaction.^4^ Retained lens fragments often trigger an inflammatory cascade, manifesting as anterior uveitis, vitritis, or secondary glaucoma, typically within days to weeks post-surgery.^4^ Prolonged delay in referral exacerbates these risks, as chronic intraocular inflammation can eventually progress to atypical posterior segment complications, such as epiretinal membrane formation or chronic macular changes.^5^ Notably, cases left untreated for over 2 months are exceptionally rare outside special circumstances. In this case, the prolonged management window was a direct consequence of the 2024 South Korean medical crisis, during which a nationwide mass resignation of trainee physicians paralyzed academic tertiary referral centers, drastically bottlenecking their capacity to accept and treat transfer patients. Extensive retinal vasculitis remains exceptionally rare in this context. We report a novel case of painful panuveitis combined with extensive retinal arteritis arising from delayed referral after retained lens fragments during cataract surgery. This presentation highlights the critical need for early vitreoretinal intervention to avert such severe, vision-threatening inflammation and vascular change.

## 2 Case report

A 61-year-old female presented to our tertiary center with a 2-week history of escalating ocular pain and conjunctival injection in her right eye. She had undergone cataract surgery at a local clinic two months prior but was not informed of any intraoperative complications such as posterior capsule rupture or retained lens fragments. Notably, her referral to a vitreoretinal specialist was significantly delayed by two months due to the 2024 Korean medical crisis, which severely restricted the capacity of regional tertiary hospitals to accept emergency cases. This necessitated an inter-provincial transfer from Chungcheong-do to northern Gyeonggi-do for definitive management. Because of this chaotic, multi-regional transfer process, the patient arrived without any official referral notes or primary operative records, rendering a direct review of her initial surgical logs unavailable.

**Fig. 1:**
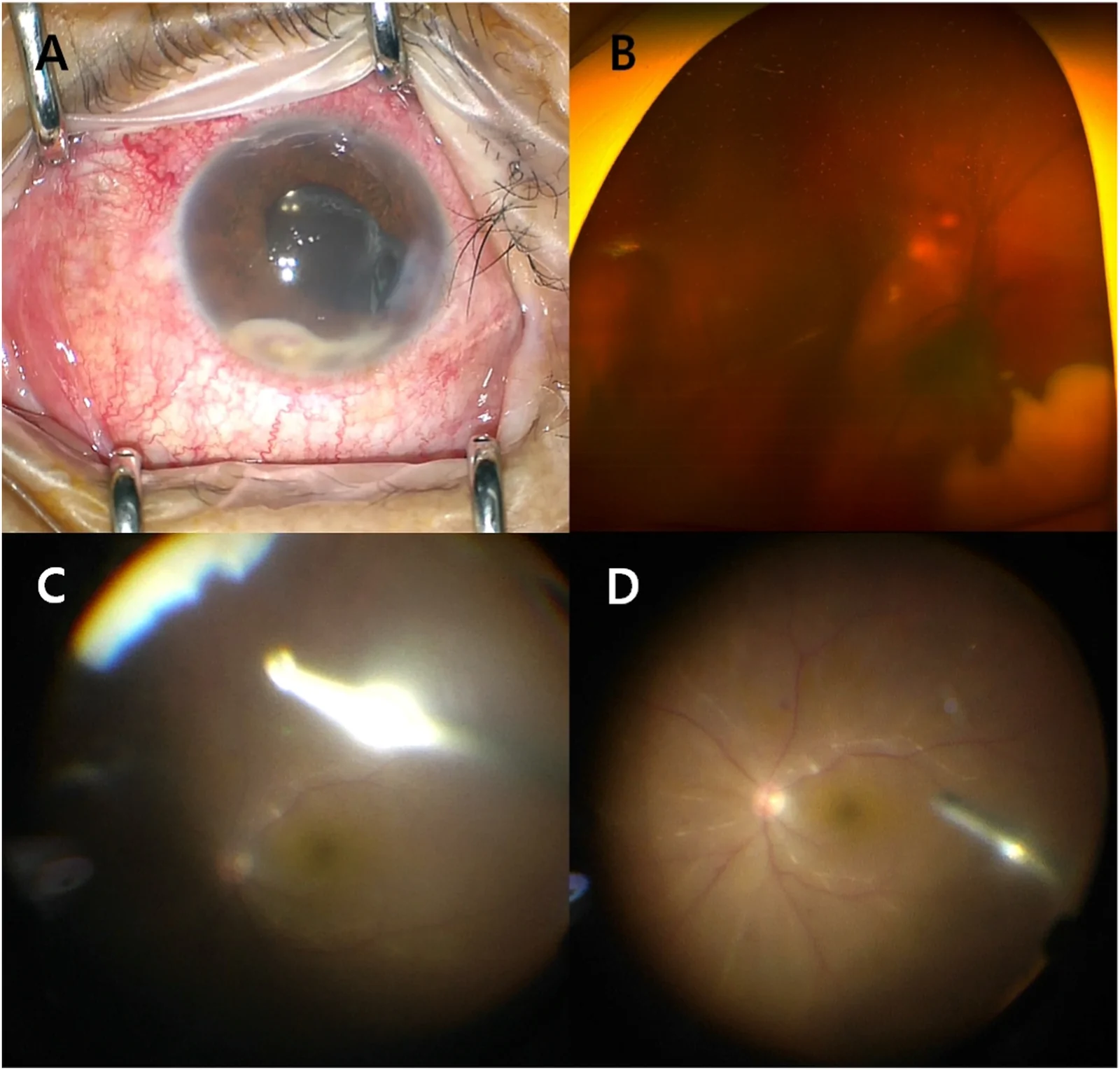
**Preoperative and intraoperative photographs of the right eye**. (A) Preoperative anterior segment photograph showing diffuse conjunctival injection with hypopyon, and iris incarceration with dragging at the main corneal incision site of the previous cataract surgery. (B) Preoperative ultra-widefield fundus photograph demonstrating severe vitreous opacity with a dimly visible optic disc and an indistinct whitish mass-like lesion in the inferonasal retinal periphery. (C) Surgeon view during pars plana vitrectomy video illustrating a dislocated retained lens particle from the previous cataract surgery positioned at the vitreous near the inferonasal retinal periphery, observed after core vitrectomy and removal of vitreous opacity. (D) Surgeon view during vitrectomy video illustrating extensive retinal arteritis with arterial sheathing and granular peri-arterial precipitates involving most retinal arteries.

Upon initial presentation, her visual acuity was reduced to hand motion, with an intraocular pressure (IOP) of 34 mmHg. Slit-lamp examination revealed severe panuveitis with a 1.5 mm hypopyon (Fig. 1A), while fundus visualization was precluded by dense vitreous opacities (Fig. 1B). These findings raised high suspicion for postoperative endophthalmitis or secondary panuveitis due to retained lens fragments.

Emergency 25-gauge pars plana vitrectomy (PPV) was performed. Anterior hypopyon was evacuated with irrigation-aspiration through the existing corneal incision, followed by synechiolysis of iris incarceration at the incision site. Four iris retractors were applied for adequate pupillary dilation. Vitreous sampling was obtained prior to connecting the infusion line, followed by core vitrectomy. The obtained vitreous specimens were sent for comprehensive microbiological analysis, including Gram staining and cultures, which subsequently yielded negative results for both bacterial and fungal organisms. Following core vitrectomy and clearance of the vitreous haze, retained lens particles from the previous cataract surgery—positioned at the vitreous near the inferonasal retinal periphery—were identified (Fig. 1C) and successfully removed by Ocutome® lensectomy (Alcon Laboratories, Inc.). Intraoperatively, a striking appearance of extensive retinal arterial sheathing—characterized by opaque, whitish vascular walls and granular peri-arterial precipitates —was observed (Fig. 1D). Intravitreal antibiotics (ceftazidime 2.25 mg/0.1 mL and vancomycin 1 mg/0.1 mL) were administered.

Postoperatively, systemic steroids were withheld for one week with oral antibiotics to rule out infectious endophthalmitis. At the one-week visit, corneal edema, anterior inflammation, and vitreous haze showed improvement; oral prednisolone (0.5 mg/kg/day) was then initiated for 1 week and tapered over 2 months.

**Fig. 2:**
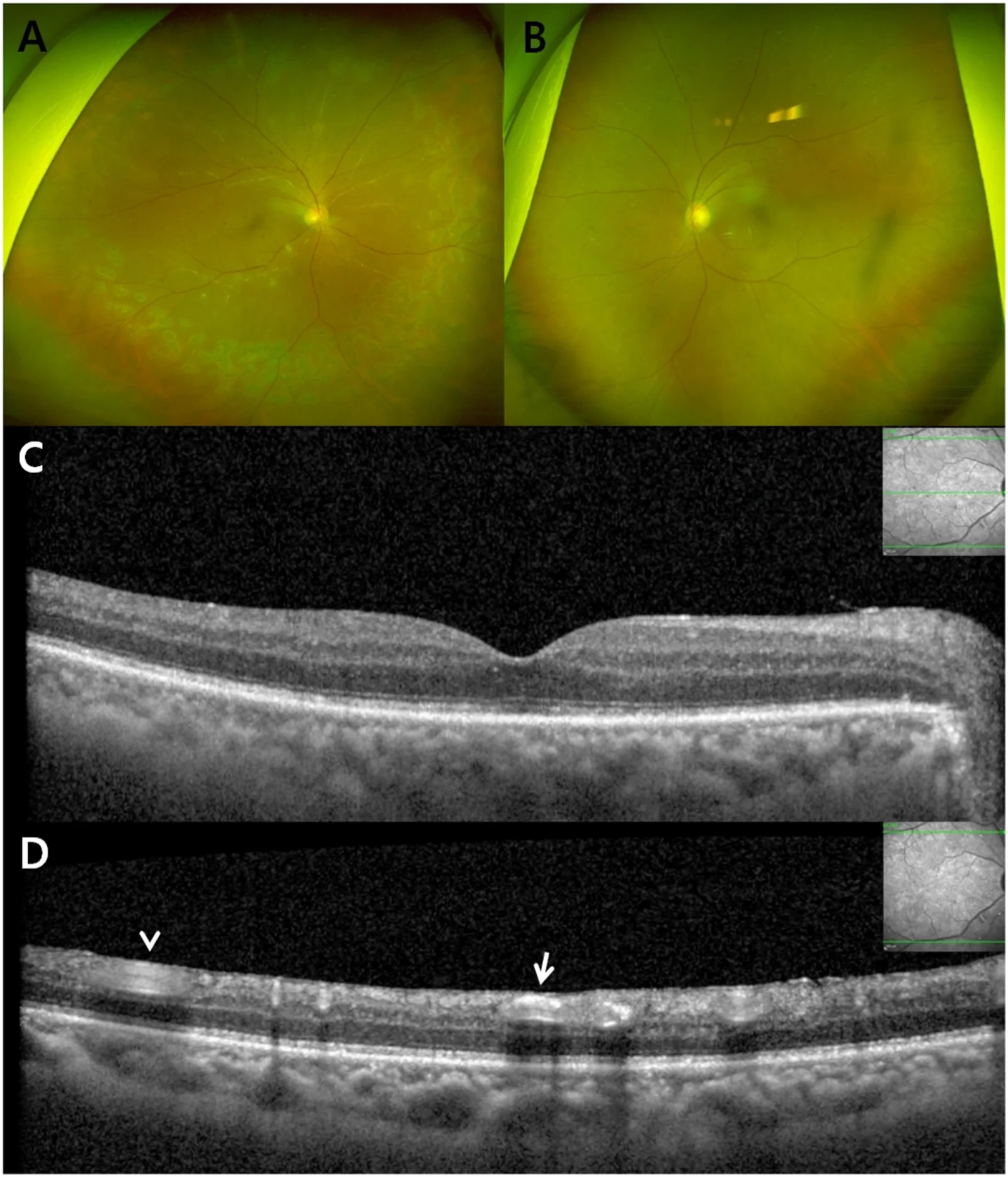
**Postoperative ultra-widefield fundus photographs and optical coherence tomography (OCT) images**. (A) Ultra-widefield fundus photograph of the right eye three weeks after surgery, showing persistent extensive retinal arteritis. (B) Ultra-widefield fundus photograph of the left eye revealing a few dot hemorrhages consistent with nonproliferative diabetic retinopathy, without extensive arteritis as seen in the right eye. (C) OCT scan of the foveal region demonstrating structurally preserved retinal architecture. (D) OCT cross-section of the superotemporal retinal arcade vessels, where the vein appears unaffected (white arrowhead) but the artery at the site corresponding to extensive arterial sheathing on fundus photography exhibits an irregular hyperreflective thickened wall ring (white arrow).

By postoperative week 3, visual acuity had recovered to 0.5 with sufficient media clarity for fundus examination. Ultra-widefield fundus photography of the right eye revealed persistent extensive retinal arteritis (Fig. 2A), while the left eye showed only a few dot hemorrhages consistent with nonproliferative diabetic retinopathy without comparable vascular changes (Fig. 2B).

**Fig. 3:**
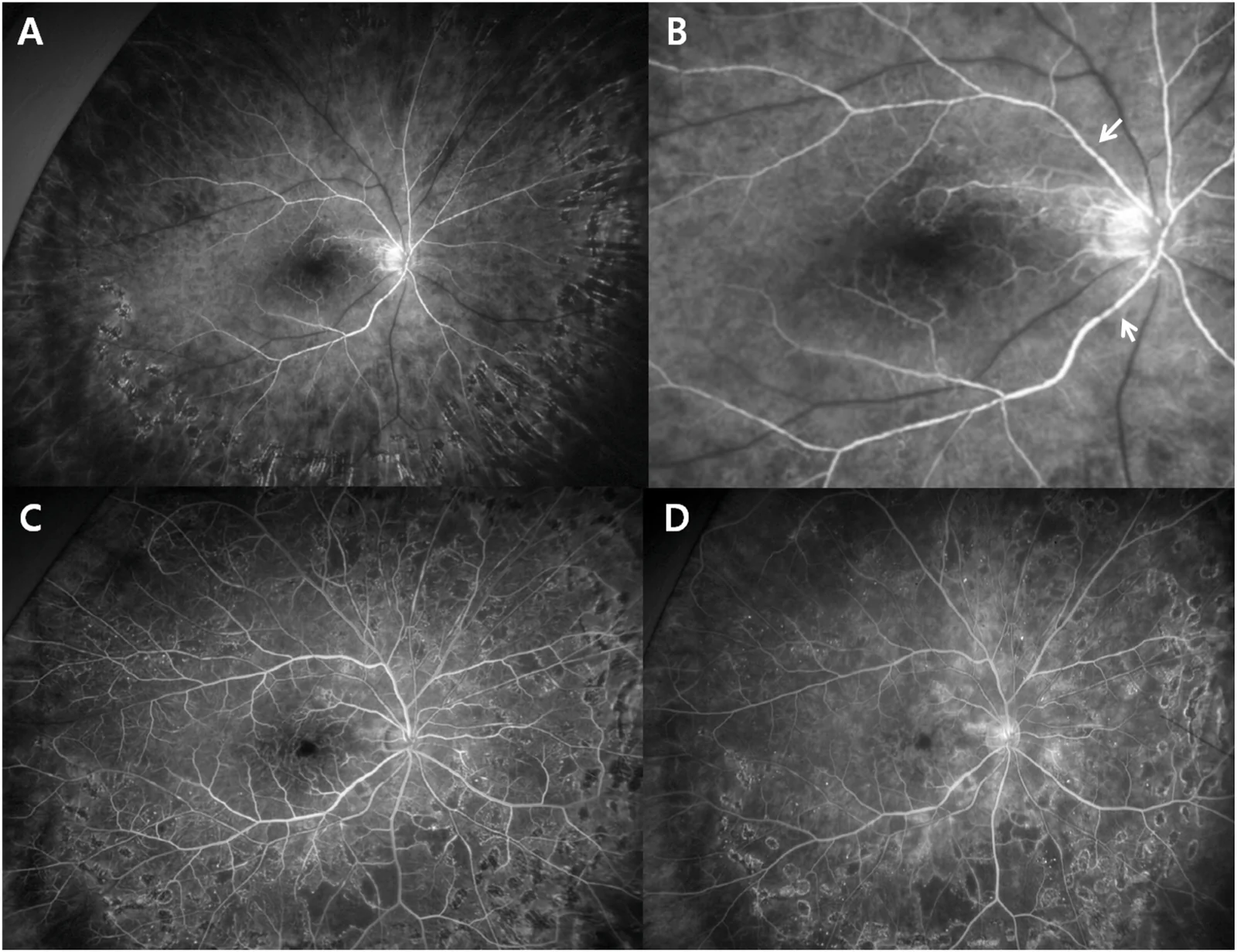
**Ultra-widefield fluorescein angiography (FA) images of the right eye three weeks after surgery**. (A) Arterial phase showing no arm-to-retina time delay and absence of retinal arterial occlusion. (B) Magnified view of the foveal region in the arterial phase revealing arterial beading (white arrows, distinct from venous beading seen in diabetic retinopathy). (C) Venous phase demonstrating capillary non-perfusion areas. (D) Late phase showing capillary leakage consistent with diabetic retinopathy, but no definite arterial leakage.

Subsequently, fluorescein angiography (FA) demonstrated segmental arterial beading and luminal stenosis without occlusion, peripheral non-perfusion, and vascular wall staining (Fig. 3A–D). Concomitant optical coherence tomography (OCT) through affected vessels revealed hyperreflective, ring-like arterial wall thickening consistent with inflammatory transmural infiltration, while the adjacent retinal vein appeared normal (Fig. 2D). At the 6-month follow-up, phacoantigenic inflammation and IOP remained well-controlled with stable visual acuity of 0.5.

## 3 Discussion

We report a rare and severe clinical progression of retained lens fragments leading to painful panuveitis with hypopyon and extensive retinal arteritis, which was objectively confirmed via OCT and FA. This atypical presentation was directly associated with an unprecedented two-month management delay driven by the 2024 South Korean medical crisis, which critically paralyzed tertiary academic referral centers. While retained lens fragments occurs approximately in 0.5-5% of cataract surgeries and triggers phacoantigenic uveitis from released lens proteins, manifesting as vitritis, glaucoma, or corneal decompensation if not addressed within days,^3^^,^^4^ our findings demonstrate that prolonged chronicity can escalate the inflammatory response into an extensive, transmural retinal arteritis mimicking endophthalmitis.

Prior literature supports vascular complications from prolonged lens retention. Besen et al. reported intraoperative periphlebitis and periarteritis directly beneath retained fragments after two weeks' neglect, with intense vitritis akin to our initial haze.^6^ Similarly, in a 69-year-old woman, vitrectomy after two months disclosed branch retinal artery occlusion-like ischemia and arteritis, confirming chronic antigen exposure drives endothelial damage and non-perfusion.^7^

In 2024, a major policy impasse between the South Korean government and the medical community led to a nationwide mass resignation of trainee physicians, resulting in a substantial reduction in the available clinical workforce across the country. During this period, many academic referral centers, including tertiary ophthalmic facilities, faced critical staffing shortages that disrupted standard referral pathways and compromised timely postoperative emergency care. In the present case, the total absence of a formal referral note and the unprecedented two-month delay in managing the retained lens fragments were heavily exacerbated by this systemic healthcare disruption. This starkly highlights how macroeconomic health policy crises can directly impair continuity of care, leading to atypical chronicity and compromised postoperative patient safety.

Distinguishing our presentation, urgent PPV with lensectomy resolved sterile inflammation (negative cultures), unmasking vasculitis only after vitritis cleared—unlike infectious endophthalmitis or vancomycin toxicity scenarios. In addition to bacterial infections, severe intraocular inflammation presenting with atypical retinal vasculitis requires careful differential diagnosis against viral etiologies, such as acute retinal necrosis, where prompt antiviral therapy could theoretically optimize clinical outcomes. However, an active viral infection was highly improbable in our patient given the prolonged, two-month latent period following the initial cataract procedure and the definitive presence of dislocated lens material. This non-infectious, sterile etiology was further supported by the complete absence of bacterial or fungal growth on microbiological analysis, and the fact that dramatic clinical resolution was achieved solely through the surgical clearance of antigenic lens material without any antiviral intervention. Therefore, the 2-month chronicity of lens protein exposure likely shifted the immune response from acute endophthalmitis to a chronic, phacoantigenic vasculopathy affecting the arterial wall structure, as evidenced by the arterial sheathing with granular peri-arterial precipitates and OCT-confirmed transmural infiltration. Final visual recovery to 0.5 underscores timely surgery's value despite delay.

In conclusion, cataract surgeons must promptly communicate complications of retained lens fragments to tertiary vitreoretinal centers to enable rapid secondary intervention; each day of delay intensifies inflammation, risking progression to panuveitis, retinal vasculitis, and irreversible vision loss. Within medical systems with less defined or disrupted referral patterns, contingency protocols prioritizing such cases remain essential to avert devastating posterior sequelae.

## Patient consent

Each study subject provided signed informed consent.

## Authorship

All authors attest that they meet the current ICMJE criteria for Authorship.

## Funding

No funding or grant support

## Declaration of competing interest

The authors declare that they have no known competing financial interests or personal relationships that could have appeared to influence the work reported in this paper.
